# A Case of Early Diagnosis of Turner Syndrome in a Neonate

**DOI:** 10.7759/cureus.16733

**Published:** 2021-07-29

**Authors:** Fatima Hemani, Sana Niaz, Vikram Kumar, Sheharyar Khan, Erum Choudry, Syed Rehan Ali

**Affiliations:** 1 Pediatrics, Indus Hospital & Health Network, Karachi, PAK; 2 Neonatology, Indus Hospital & Health Network, Karachi, PAK; 3 Family Medicine, Baqai Medical University, Karachi, PAK; 4 Dentistry, The Indus Hospital, Indus Hospital Research Center, Karachi, PAK

**Keywords:** sexual infantilism, turner syndrome, karyotype, hypogonadism, short stature

## Abstract

Turner syndrome (TS), or Bonnevie-Ullrich syndrome, also known as congenital ovarian hypoplasia syndrome, is the most common sex chromosome abnormality in females in approximately 1 in 2000 live birth. It occurs when the X chromosome is partially or completely missing in females caused by monosomy or structural abnormalities of the X chromosome. It is mainly diagnosed in late childhood or adolescent age and rarely identified during the neonatal period. It is characterized by short stature, webbed neck, lymphedema of extremities, widely spaced-out nipples, and cubital valgus. Early diagnosis of TS allows for appropriate and timely initiation of therapy with comprehensive care. We report a case of a neonate presented with the complaint of edema of feet since birth and syndromic features. TS was diagnosed by the chromosomal analysis, which demonstrated a gene karyotype of 46.X,i(X)(q10){20}.

## Introduction

In 1938, an Oklahoma physician, Dr. Henry Turner, distinguished several cases of similar appearance, that is, women with short stature, webbing of neck, cubitus valgus, and sexual infantilism. He named the condition Turner syndrome (TS) [1]. In 1959, it was identified that the disease is caused by monosomy or structural defects of the X chromosome [2]. TS is one of the most common disorders caused by chromosomal aneuploidy. In 45% of cases, it is due to 45XO monosomy, while in other cases, it is caused by a chromosomal abnormality or mosaicism of 45X [3]. In 98% of cases, it is widely associated with spontaneous abortions, while the conditions affect approximately 1 in 2000 to 2500 live female birth. Unfortunately, only 20% of cases are diagnosed at birth, and the rest are identified during childhood or prepubertal stages [4-5].

It is characterized by a combination of physical and cytogenetic features. The clinical manifestation of TS can range widely among the affected individuals. Gonadal dysgenesis and decreased growth are classical features in TS; many other organ systems are also affected by varying patterns and at different periods of life. Infants with TS have a high rate of growth failure at birth. However, the pattern and time of onset of growth and development deficiency are undetermined [2, 6].

An eight-day-old neonate who was presented with typical TS features has been reported here.

## Case presentation

An eight-day-old female neonate was referred to the outpatient department of neonatology at the Indus Hospital & Health Network, Karachi, Pakistan, with the complaint of dysmorphic features and edema of both feet since birth. Medical history revealed that she was born via emergency cesarean section at the 38th week due to decreased fetal movements, but there was no complication during or after delivery. The mother's antenatal scans were normal. There was no history of fetal distress, delayed crying, lethargy, feeding difficulty, fever, or fits, no family history of recurrent miscarriages, infantile deaths, or any genetic diseases. No family member had similar facial features. When taking a family history, it was revealed that the parents had a consanguineous marriage.

**Figure 1:**
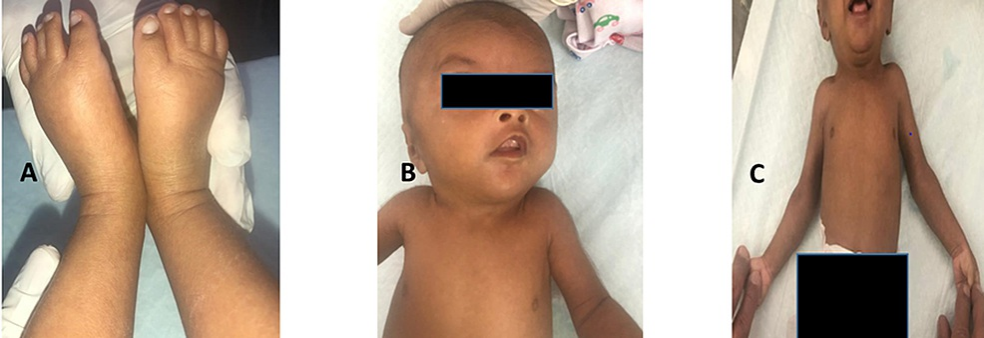
Clinical photographs of the neonate showing features of Turner syndrome (A: bilateral pedal edema, B: webbed neck, C: wide-spaced nipples).

The baby had normal vitals with a birth weight of 2.7 kg and 48 cm height on physical examination. She was active, with intact sucking, moro, and grasping reflexes. In addition, there was bilateral non-pitting edema of feet extending up to ankles, webbed neck, wide-spaced nipples, an extra thin peduncle-like appendage on the right hand, cubital valgus, and a small lower jaw (Figure 1).

**Figure 2:**
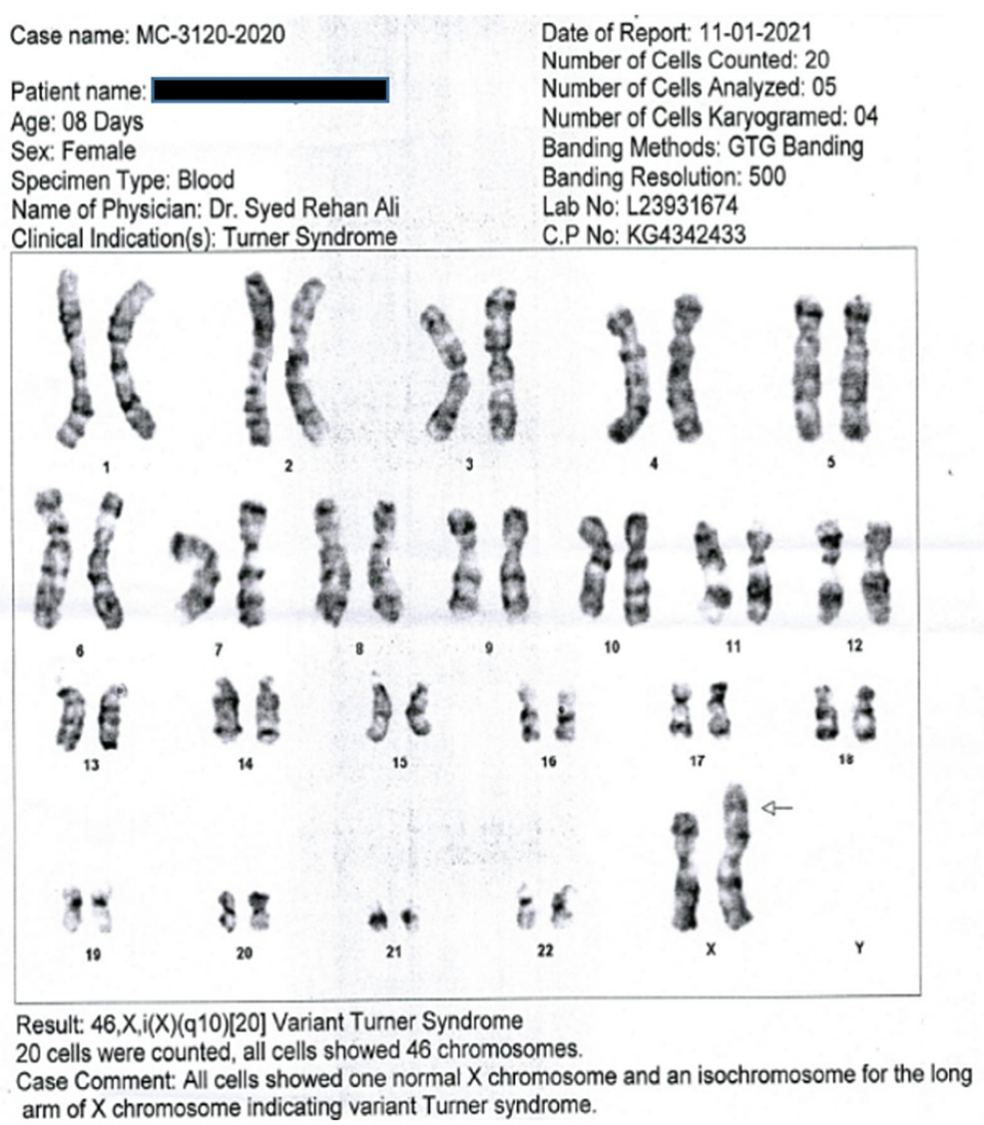
Cytogenetic report showing a gene karyotype of 46.X,i(X)(q10){20}.

For confirmatory diagnosis, chromosomal karyotyping, echocardiography, complete blood count (CBC), serum electrolytes, serum thyroid-stimulating hormone (TSH), ultrasound kidney, ureter, and bladder (KUB), and brain were performed. Chromosomal analysis of the patient showed a TS variant of 46.X, i(X)(q10){20} (Figure 2).

**Figure 3:**
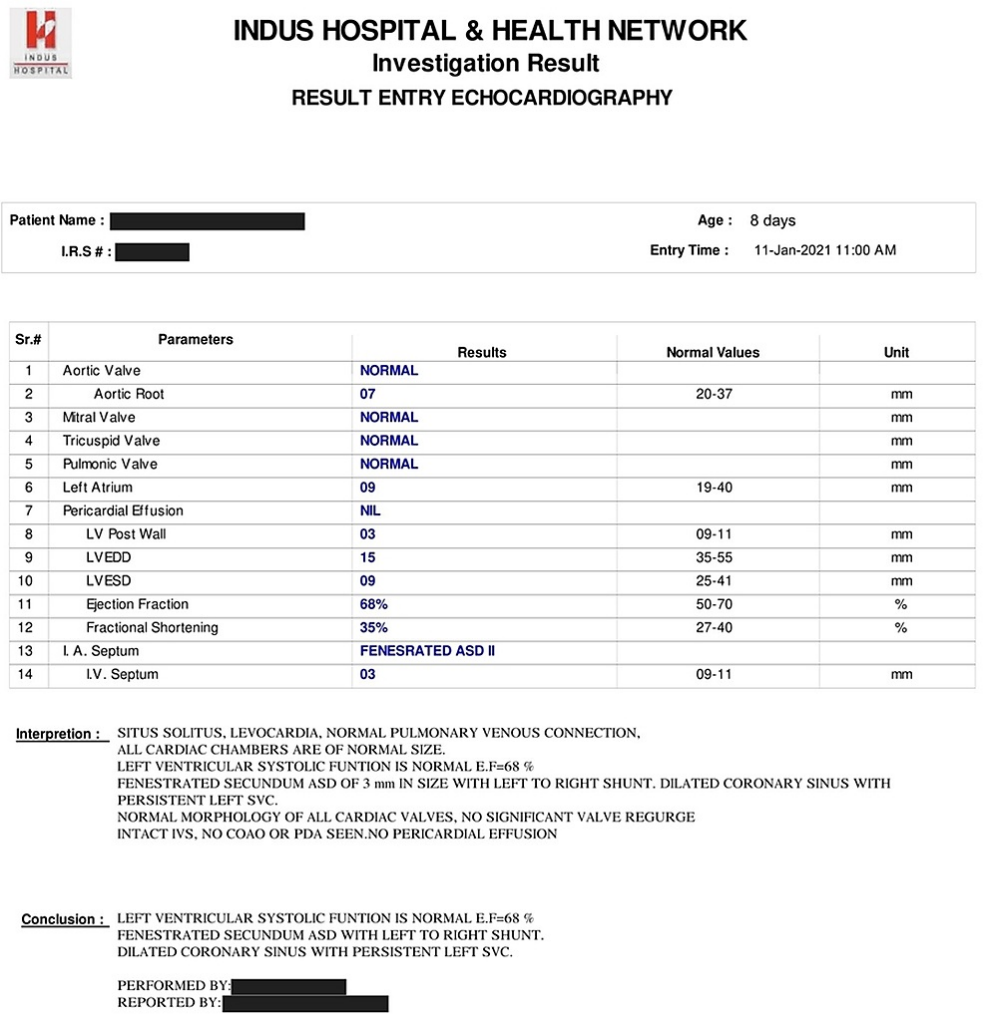
Echocardiography report showing cardiac anomaly. LV postal wall: Left ventricular postal wall; LV EDD: Left ventricular end-diastolic dimension; LV ESD: Left ventricular end-systolic dimension; IA septum: Interatrial septum; IV septum: Interventricular septum.

Echocardiography showed a fenestrated secundum atrial septal defect (ASD) of 3 mm with a left-to-right shunt and dilated coronary sinus with persistent left superior vena cava (PLSVC) (Figure 3). The rest of the routine labs, including electrolytes, TSH, U/S brain, and KUB, were also normal.

After the careful evaluation and disease diagnosis, parents were counseled about the baby's condition and were advised to follow up after three months. Additionally, psychological support was provided to the parents to help them accept and understand their child's disease. The first follow-up was conducted in March 2021, where the child was assessed for her progress and was advised to follow up after every six months to monitor the development and growth of the child.

## Discussion

The human genome has 46 chromosomes, including 22 pairs of autosomes and the X and Y chromosomes. Women have two complete X chromosomes when they are born. Turner's patients typically have 44 autosomes plus one X chromosome. In TS, girls either miss one X chromosome (45X) or a part of it. While in mosaicism, parts of the body have normal cells with two X chromosomes (46XX), while others have one X chromosome missing (45X) [3, 6].

TS is a complex medical disorder characterized by physical, psychological, and oral features. TS was initially described as a triad of cubitus valgus, infantilism, and facial features including a short, broad neck with webbing, low-set ears, and down-slanted palpebral fissures with epicanthal folds [1]. TS is also characterized by congenital lymphedema of feet and hands, short stature, low posterior hairline, widely spaced nipples, and primary amenorrhea. It is also associated with congenital renal (horseshoe kidney), cardiovascular (coarctation of the aorta or bicuspid aortic valve), and thyroid disease [4]. In addition, many features in the oral cavity typical of the high-arched palate, hypoplastic mandible, thin enamel and decreased amount of dentin, tooth mobility, and periodontal pockets, prematurely erupted teeth, and various malocclusions [6]. These clinical presentations can vary depending on karyotyping. Some patients with mosaicism only present as short stature and primary amenorrhea without dysmorphic features, but most females with TS have ovarian failure [7]. As a consequence of ovarian failure, females with TS have hyper gonadotropic hypogonadism. Nearly 30% of females with TS spontaneously begin puberty, but only around 4% of them reach menarche [8]. Patients with TS are at increase risk of psoriasis, juvenile idiopathic arthritis, type 1 diabetes, adrenocortical insufficiency, osteoporosis, and inflammatory bowel disease. Human leukocyte antigen genes analysis is of vital importance, as patients with TS are highly susceptible to autoimmune disease [9-10]. The overall mortality rate for patients with TS is higher than that for normal individuals because of the higher incidence of cardiovascular disease and autoimmune diseases [10]. Karyotyping is the gold standard test for the diagnosis of TS. Individuals with classic XO karyotype usually present during the neonatal period, while individuals with mosaic XO present later in adulthood [11]. Amniocentesis or chorionic villus sampling conducted in pregnancy can also help to diagnose TS during the prenatal period. Additionally, the presence of thickening of the nuchal folds, renal anomalies, cystic hygroma, or left-sided cardiac anomalies on fetal USG may also raise prenatal suspicion for TS [12]. The primary treatments for individuals affected by TS are estrogen and growth hormone therapies. Many females with TS require hormone replacement therapy to ensure the development of secondary sexual characteristics, begin puberty, or reach menarche. Growth hormone therapy is also needed for bone development and to increase height (Table 1) [13]. Table 1 shows the different treatment options available for TS.

**Table 1:** Treatment options available for Turner syndrome.

Treatment of Turner syndrome	Indications and Importance	References
Growth hormone therapy	Growth hormone therapy can increase the height of patients. Lifelong height is related to age, dose, and duration of treatment. Combination therapies are recommended than therapy with growth hormone alone.	[14-15]
Estrogen replacement therapy	A low dose of estrogen replacement therapy is recommended at the age of 12 to develop the uterus and secondary sexual characteristics and help improve liver function, cognitive function, and quality of life.	[13, 15]
Oxymetholone treatment	A marked effect can be seen when used in combination with growth hormone. However, Adverse reactions may occur, so this treatment should be used with caution.	[16]
Calcium and Vitamin D supplementation	To strengthen the bones and prevent them from fractures by increasing bone mineral density.	[13]
Other methods	Traditional Chinese medicine treatment (Liuwei Dihuang pills).	[15]
Psychosocial support	Providing training focused on general coping strategies, adaptive skills, stress management, self-esteem improvement, and practicing an active lifestyle with regular sports can be extremely beneficial.	[15]

Prompt diagnosis with early intervention can help TS patients achieve average physical growth. In our case, the baby was presented with typical features of TS, which were first noticed by the parents. Due to a high suspicion of TS, her sample was sent for chromosomal karyotyping. Fortunately, this child was diagnosed exceptionally early, and thus the physicians took the necessary measures to preserve the patient's quality of life. The parents were counseled in detail regarding the child's condition to ensure a satisfactory environment for optimal growth. This timely treatment is expected to positively impact the child's life, psychologically, physically, and socially.

## Conclusions

Most of the TS cases are diagnosed in pubertal years due to stunted growth; hence, it is essential to diagnose these cases during the neonatal period to develop appropriate hormonal therapy for development, growth, and pubertal induction. It is also important to diagnose TS early so that screening for congenital heart disease, horseshoe kidney, and hypothyroidism commonly associated with TS can be detected before the presentation of symptoms. Clinical and non-clinical parts of TS in younger patients should be continuously addressed by a multidisciplinary team of clinicians and health care professionals, having expertise with such patients. They should focus on patients' mental, reproductive, and physical growth to minimize complications associated with TS. Regular follow-ups and support group therapies can help TS females lead a healthy and relatively normal life.
